# Delay of Morphine Tolerance by Palmitoylethanolamide

**DOI:** 10.1155/2015/894732

**Published:** 2015-03-22

**Authors:** Lorenzo Di Cesare Mannelli, Francesca Corti, Laura Micheli, Matteo Zanardelli, Carla Ghelardini

**Affiliations:** Dipartimento di Neuroscienze, Psicologia, Area del Farmaco e Salute del Bambino-Neurofarba-Sezione di Farmacologia e Tossicologia, Università di Firenze, Viale Pieraccini 6, 50139 Florence, Italy

## Abstract

In spite of the potency and efficacy of morphine, its clinical application for chronic persistent pain is limited by the development of tolerance to the antinociceptive effect. The cellular and molecular mechanisms underlying morphine tolerance are complex and still unclear. Recently, the activation of glial cells and the release of glia-derived proinflammatory mediators have been suggested to play a role in the phenomenon. *N*-Palmitoylethanolamine (PEA) is an endogenous compound with antinociceptive effects able to reduce the glial activation. On this basis, 30 mg kg^−1^ PEA was subcutaneously daily administered in morphine treated rats (10 mg kg^−1^ intraperitoneally, daily). PEA treatment significantly attenuated the development of tolerance doubling the number of days of morphine antinociceptive efficacy in comparison to the vehicle + morphine group. PEA prevented both microglia and astrocyte cell number increase induced by morphine in the dorsal horn; on the contrary, the morphine-dependent increase of spinal TNF-*α* levels was not modified by PEA. Nevertheless, the immunohistochemical analysis revealed significantly higher TNF-*α* immunoreactivity in astrocytes of PEA-protected rats suggesting a PEA-mediated decrease of cytokine release from astrocyte. PEA intervenes in the nervous alterations that lead to the lack of morphine antinociceptive effects; a possible application of this endogenous compound in opioid-based therapies is suggested.

## 1. Introduction

Opioids remain an integral part of clinical pain management [1]. Although often successful in acute settings, long-term use of opioids may be accompanied by waning levels of analgesic response not readily attributable to advancing underlying disease, necessitating dose escalation to manage pain. Analgesic tolerance has been invoked to explain such declines in opioid effectiveness over time. This undesirable manifestation, along with other adverse effects caused by escalating doses (e.g., oversedation, respiratory depression, and constipation), significantly decreases quality of life in patients with chronic pain [2].

Lines of evidence have demonstrated that multiple factors are known to be involved in morphine tolerance [3], mainly involving neuronal mechanisms of adaptation and sensitization. On the other hand, chronic morphine treatment activates spinal and cortical glial cells [4–7] which contribute the development of antinociceptive tolerance [6, 8]. Direct and indirect morphine-evoked signals [7] produce microglia and astrocyte changes [9] ultimately resulting in increased production of many substances such as free radicals, nitric oxide, proinflammatory cytokines and chemokines, prostaglandins, complement proteins, neurotoxins, neurotrophic factors, and excitatory amino acids which actively opposes the analgesic effects of morphine and contributes to the development of tolerance [10, 11]. Moreover, pharmacological glial inhibition decreases morphine-induced cytokine release and attenuates tolerance [7]. Administration of the glial metabolic inhibitor fluorocitrate has been found to attenuate the development of morphine tolerance [6]. Minocycline, propentofylline, and pentoxifylline reduced glial cell activation and significantly blocked the development of morphine tolerance in naive mice, as well as in a model of neuropathic pain [5, 12, 13]. Lu et al. [14] showed that patients receiving pentoxifylline exhibited longer patient-controlled analgesia trigger times in the presence of attenuated perioperative cytokine response and required less morphine consumption. However the side effects of these compounds limit their prolonged use in persistent pain conditions [15].

*N*-Palmitoylethanolamine (PEA), the endogenous amide between palmitic acid and ethanolamine, belongs to the family of fatty acid ethanolamides (FAEs), a class of lipid mediators. PEA exerts antinociceptive effects in several animal models [16, 17]. Its safety and efficacy were shown in a variety of clinical trials focused on pain state treatment: diabetic neuropathy, carpal tunnel syndrome, dental and temporomandibular joint pain, and arthritic, postherpetic, and chemotherapy-induced neuropathic pain [18, 19]. Moreover, PEA protects nervous tissue in neuropathic conditions [20], prevents neurotoxicity and neurodegeneration [21, 22], and inhibits peripheral inflammation and mast cell degranulation [23]. Further, PEA reduced the activation of microglia and astrocytes [24]. PEA normalized spinal microglia and astrocyte activation in the rat model of inflammatory pain induced by formalin [25] as well as after spinal cord trauma in mice [26]. Treatment with PEA reduced microglial activation and the number of astrocytes in the model of Parkinson's disease induced by 1-methyl-4-phenyl-1,2,3,6-tetrahydropyridine (MPTP) [27] and counteracts reactive gliosis after *β*-amyloid peptide injection in rat brain [28].

Based on the hypothesis that the glial cell modulator PEA may influence the development of morphine tolerance, the antinociceptive effect of repeated treatment with the alkaloid was evaluated overtime during PEA administration.

## 2. Material and Methods

### 2.1. Animals

Male Sprague-Dawley rats (Harlan, Varese, Italy), weighing 200–250 g at the beginning of the experimental procedure, were used for all the experiments. Animals were housed in CeSAL (Centro Stabulazione Animali da Laboratorio, University of Florence) and used no earlier than one week after their arrival. Four rats were housed per cage (size 26 × 41 cm); animals were fed with standard laboratory diet and tap water* ad libitum* and kept at 23 ± 1°C with a 12 h light/dark cycle, light at 7 a.m. All animal manipulations were carried out according to the European Community guidelines for animal care (DL 116/92, application of the European Communities Council Directive of 24 November 1986; 86/609/EEC). The ethical policy of the University of Florence complies with the Guide for the Care and Use of Laboratory Animals of the US National Institutes of Health (NIH Publication number 85-23, revised 1996; University of Florence assurance number: A5278-01). Formal approval to conduct the described experiments was obtained from the Animal Subjects Review Board of the University of Florence and the research was authorized by the Italian Ministry of Health (Decree 54/2014-B). All efforts were made to minimize animal suffering and to reduce the number of animals used.

### 2.2. Pharmacological Treatments

Micronized PEA (Epitech, Padova, Italy) was dissolved in PEG and Tween 80 2 : 1 (Sigma-Aldrich, Milan, Italy) and kept overnight under gentle agitation with a microstirring bar. Before injection, sterile saline was added so that the final concentrations of PEG and Tween 80 were 20 and 10% v/v, respectively. Drug was injected daily (9 a.m., from day 1 to day 11) subcutaneously (s.c.) in a dose of 30 mg kg^−1^. Morphine (S.A.L.A.R.S., Como, Italy) was dissolved in sterile saline and injected daily (2 p.m., from day 1 to day 11) intraperitoneally (i.p.) in a dose of 10 mg kg^−1^. Behavioral measurements were performed immediately before and 30 min after morphine administration. Dosages were chosen on the basis of previous studies [20, 29, 30]. The described dosages were administered with respect to the body weight and all injections were given in a mean volume of 0.3 mL. Control animals were treated with vehicle.

### 2.3. Paw Pressure Test

The nociceptive threshold in the rat was determined with an analgesiometer (Ugo Basile, Varese, Italy), according to the method described by [31]. Briefly, constantly increasing pressure was applied to a small area of the dorsal surface of the hind paw using a blunt conical mechanical probe. Mechanical pressure was increased until vocalization or a withdrawal reflex occurred while rats were lightly restrained. Vocalization or withdrawal reflex thresholds were expressed in grams. Rats scoring below 40 g or over 75 g during the test before drug administration were rejected (25%). For analgesia measures, mechanical pressure application was stopped at 120 g [32].

### 2.4. Plantar Test

Pain thermal sensitivity was measured using a plantar test apparatus (Ugo Basile, Varese, Italy), wherein the paw withdrawal latency to a thermal stimulus was measured, as described previously [33]. The apparatus used a test unit containing a heat source that radiated a light beam. An adjustable angled mirror on the test unit was used to locate the correct targeting area on the paw. The beam source was set with an active intensity of 40%, an idle intensity of 10%, and a cut-off time of 25 s. The paw withdrawal latency comprised the time from the start of the beam light until the animal withdrew the paw from the heat stimulus (reaction time was measured to 0.01 s). An acrylic six-chamber container was used to separate the rats that were placed on the glass base. The baseline paw withdrawal latency values were close to 10 s when the current parameters were used. Measurements were taken in duplicate at least 1 min apart, and the average was used for statistical analysis. Behavioural responses of both left and right paws were measured.

### 2.5. Immunofluorescence Staining

On days 6 and 11, rats were sacrificed; the L4/L5 segments of the spinal cord were exposed from the lumbovertebral column via laminectomy and identified by tracing the dorsal roots from their respective DRG. According to [34–36], formalin-fixed cryostat sections (7 *μ*m) were washed 3× with phosphate-buffered saline (PBS) and 0.3% Triton X-100 for 5 min and then were incubated, at room temperature, for 1 h in blocking solution (PBS, 0.3% Triton X-100, and 5% albumin bovine serum; PBST). Slices were incubated overnight at 4°C in PBST containing rabbit primary antisera. The primary antibody used was directed against ionized calcium binding adapter molecule 1 (Iba1; rabbit, 1 : 1000; Wako, Richmond, VA, USA) for microglial staining or against glial fibrillary acidic protein (GFAP; rabbit, 1 : 1000; DAKO, Carpinteria, CA, USA) for astrocyte staining. The following day slides were washed 3× with PBS and 0.3% Triton X-100 for 5 min and then sections were incubated in goat anti-rabbit IgG secondary antibody labeled with Alexa Fluor 568 (1 : 500; Invitrogen, Carlsbad, USA) and DAPI (4′,6-diamidin-2-phenylindole; 1 : 2000; Life Technologies-Thermo scientific, Rockford, IL, USA), a nuclei marker, in PBST at room temperature for 2 h, in the dark. After 3× PBS and 0.3% Triton X-100 wash for 10 min, slices were mounted using ProLong Gold (Life Technologies-Thermo scientific, Rockford, IL, USA) as a mounting medium.

Negative control sections (no exposure to the primary antisera) were processed concurrently with the other sections for all immunohistochemical studies, in order to exclude the presence of nonspecific immunofluorescent staining or cross immunostaining.

Images were acquired by using an Olympus BX63 microscope equipped with an Olympus XM10 camera and coupled to* CellSens Dimension* Software (Olympus, Milan, Italy).

Quantitative analysis of GFAP and Iba1-positive cells was performed by collecting three independent fields through a 20x 0.40NA objective in the dorsal horn of each rat spinal cord. GFAP and Iba1-positive cells were counted using the “cell counter” plugin of ImageJ (NIH, Bethesda, Maryland, USA).

### 2.6. Double Immunofluorescence Staining

To evaluate the tumor necrosis factor-*α* (TNF-*α*) expression in the dorsal horn of rat spinal cord, double immunofluorescent labeling of TNF-*α* and GFAP for astrocytes or OX42 for microglia was performed. Formalin-fixed cryostat sections (7 *μ*m) were washed 3× with PBS and 0.3% Triton X-100 for 5 min and then were incubated, at room temperature, for 1 h in PBST. To visualize TNF-*α* and microglia the primary antibodies used were directed against TNF-*α* (rabbit, 1 : 1000; Thermo scientific, Rockford, IL, USA) and OX42, a microglia marker (mouse, 1 : 150; BD Bioscience, Becton&Dickinson, New Jersey, USA). Antibodies were incubated overnight at 4°C in PBST. The following day, slides were washed 3× PBS and 0.3% Triton X-100 for 5 min and then sections were incubated in goat anti-rabbit IgG secondary antibody labeled with Alexa Fluor 568 (1 : 500, Invitrogen, Carlsbad, USA), to visualize TNF-*α*, and in goat anti-mouse IgG secondary antibody labeled with Alexa Fluor 488 (1 : 500; Invitrogen, Carlsbad, USA), to visualize microglia, and DAPI (1 : 2000, Life Technologies-Thermo scientific, Rockford, IL, USA), a nuclei marker, in PBST at room temperature for 2 h in the dark. After 3× PBS and 0.3% Triton X-100 wash for 10 min, slices were mounted using ProLong Gold (Life Technologies-Thermo scientific, Rockford, IL, USA) as a mounting medium. The same procedure was repeated to visualize TNF-*α* and astrocytes using the described antibody directed against TNF-*α* and a mouse GFAP Alexa Fluor 488 conjugated (1 : 200; Millipore, Temecula, CA, USA). Negative control sections (no exposure to the primary antisera) were processed concurrently with the other sections for all immunohistochemical studies, in order to exclude the presence of nonspecific immunofluorescent staining or cross immunostaining. Images were acquired as above. Quantitative analysis of TNF-*α* and GFAP expression or TNF-*α* and OX42 expression was performed by collecting three independent fields through a 20x 0.40NA objective in the dorsal horn of each rat spinal cord. Colocalization that can be described as the spatial overlap of two or more dyes in a multichannel image of TNF-*α* and GFAP or of TNF-*α* and OX42 was evaluated using the “JACoP” (just another colocalization plugin) plugin of ImageJ (NIH, Bethesda, Maryland, USA). Colocalization can be estimated by calculating a number of values representing the proportion of colocalized pixels. These values are called colocalization coefficients [37]. In this work we evaluated the overlap coefficient: it indicates an actual overlap of the signals and is considered to represent the true degree of colocalization [38].

### 2.7. Enzyme-Linked Immunosorbent Assay (ELISA) TNF-*α*

The dorsal horns of the spinal cord were homogenized in lysis buffer containing 50 mM Tris-HCl pH 8.0, 150 mM NaCl, 1 mM EDTA, 0.5% Triton X-100, Complete Protease Inhibitor (Roche, Milan, Italy), in ice, and centrifuged at 13,000 ×g for 15 minutes at 4°C. The protein concentration of the supernatant was quantified by BCA assay kit (Sigma-Aldrich, St. Louis, MO, USA). TNF-*α* was measured using commercially available enzyme immunoassays (rat TNF-*α* ELISA set, eBiosciences, San Diego, CA, USA) according to the manufacturer's instructions. The protein expression was normalized to the total protein amount per spinal cord and reported as pg/mg.

### 2.8. Statistical Analysis

Behavioral measurements were performed on 12 rats for each treatment carried out in 2 different experimental sets. Measurements were taken in duplicate at least 1 min apart; the responses of both left and right paws were measured. For behavioral experiments one-way analysis of variance (ANOVA) followed by Fisher's protected least significant difference procedure was used. ELISA and immunohistochemical analyses were performed on 6 rats per group. Six sections of spinal cord for each animal were evaluated. For statistical analysis, data were analyzed by one-way ANOVA followed by multiple comparisons with the Bonferroni post hoc test.

All behavioral assessments were made by researchers blinded to rat treatment. Slides from control and experimental groups were labeled with numbers so that the person performing the image analysis was blinded as to the experimental group. In addition, all images were captured and analyzed by an investigator other than the one who performed measurements to avoid possible bias. Since behavioral measurements were performed 30 min after morphine injection, different animal groups were used for paw pressure and plantar tests. ELISA and immunohistochemical analysis were performed on tissues from the same animals used for behavioral analysis. Data about the control group vehicle + vehicle are the mean of values obtained on days 6 and 11. For all the immunochemical analyses no differences were highlighted in the vehicle + vehicle group on days 6 and 11. Data were analyzed using the “Origin 7.5” software (OriginLab, Northampton, MA, USA). Differences were considered significant at *P* < 0.05.

## 3. Results

**Figure 1:**
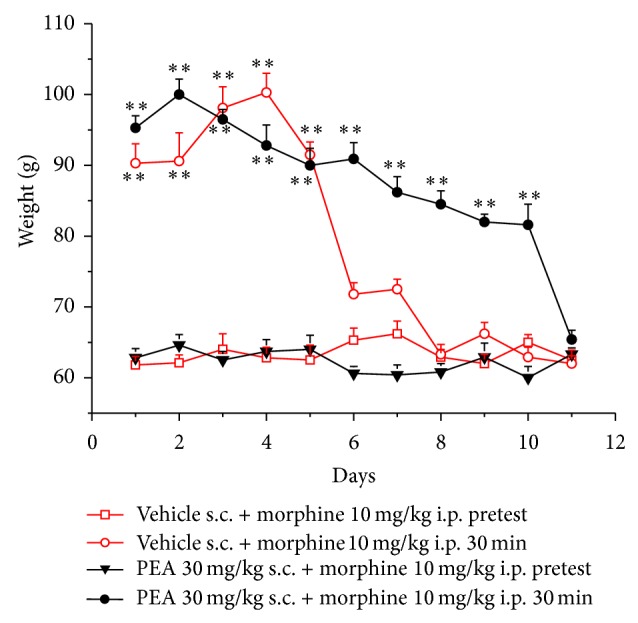
Analgesia measurement, paw pressure test. Animals were treated daily with 30 mg kg^−1^ PEA s.c. or with vehicle. The pain threshold was evaluated every day immediately before and 30 min after the injection i.p. of 10 mg kg^−1^ morphine. Each value represents the mean ± SEM of 12 rats per group, performed in 2 different experimental sets. ^**^ *P* < 0.01 versus pretest values.

**Figure 2:**
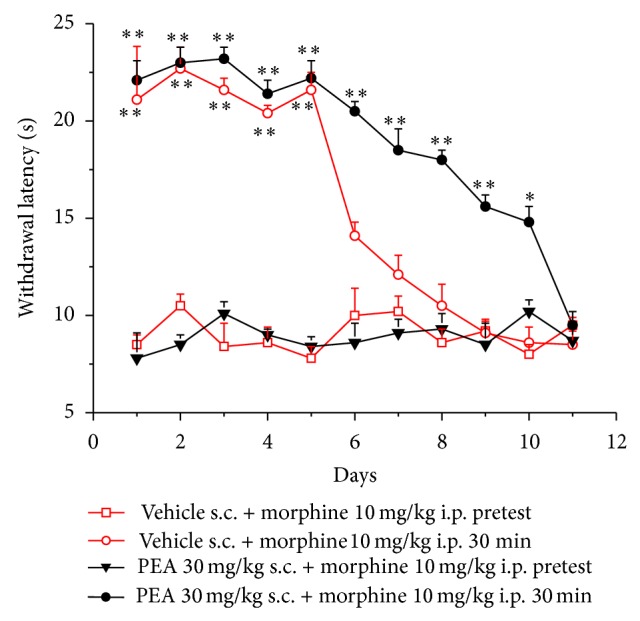
Analgesia measurement, plantar test. Animals were treated daily with 30 mg kg^−1^ PEA s.c. or with vehicle. The pain threshold was evaluated every day immediately before and 30 min after the injection i.p. of 10 mg kg^−1^ morphine. Each value represents the mean ± SEM of 12 rats per group, performed in 2 different experimental sets. ^**^ *P* < 0.01 versus pretest values.

Ten mg kg^−1^ morphine administered i.p. (30 min after injection) increased the weight tolerated on the posterior paw up to 90.3 ± 2.7 g in comparison to the threshold before treatment (pretest) of 61.8 ± 1.2 g (Figure 1). A similar effect was maintained when morphine was newly injected in the following days till day 5. The same dose was unable to significantly increase pain threshold from day 6. In the group treated with 30 mg kg^−1^ PEA s.c. (daily) the antinociceptive effect of morphine reached 95.3 ± 1.7 g (Figure 1). The efficacy of morphine was significant up to day 10 (81.6 ± 2.9 g). PEA per se did not alter the response to the paw pressure as shown by the values of pretest (before morphine injection) of the group PEA + morphine.  Figure 2 shows the results obtained with the plantar test. The withdrawal latency to a painful thermal stimulus was increased by morphine to 21.1 ± 2.7 s in comparison to the pretest value of 8.5 ± 0.5 s (vehicle + morphine group). In the presence of PEA treatment the significance of morphine-induced analgesia lasted till day 10 (14.8 ± 0.8 s).

The spinal cord was analyzed on days 6 and 11 when tolerance to the antinociceptive effect of morphine was developed in the vehicle + morphine and in the PEA + morphine group, respectively.

**Figure 3:**
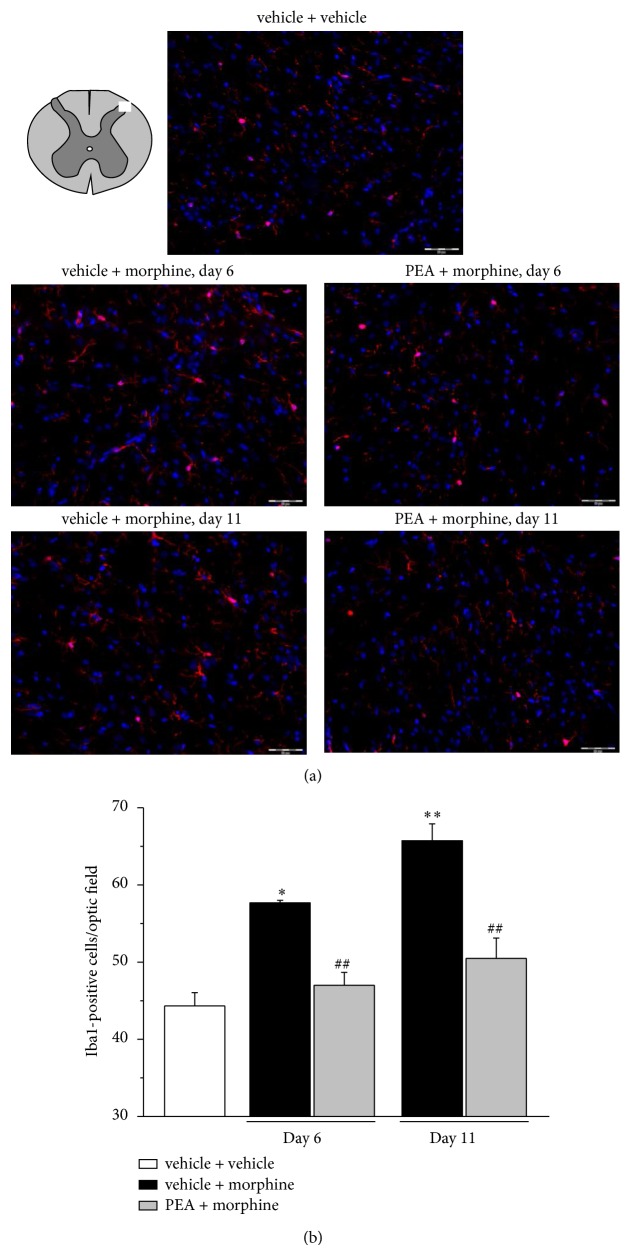
Iba1-positive cell density in the dorsal horn of the spinal cord. 30 mg kg^−1^ PEA s.c. and 10 mg kg^−1^ morphine i.p. were administered daily and immunohistochemical analysis was performed on days 6 and 11; (a) representative images of merged Iba1-labeled microglia cells (red), plus DAPI-labeled cell nuclei (blue); scale bar: 50 *μ*m. (b) Quantitative analysis of cellular density was performed evaluating 6 animals for each group. Each value represents the mean ± SEM of 6 rats per group, performed in 2 different experimental sets. ^*^ *P* < 0.05 and ^**^ *P* < 0.01 versus vehicle + vehicle; ^##^ *P* < 0.01 versus vehicle + morphine.

**Figure 4:**
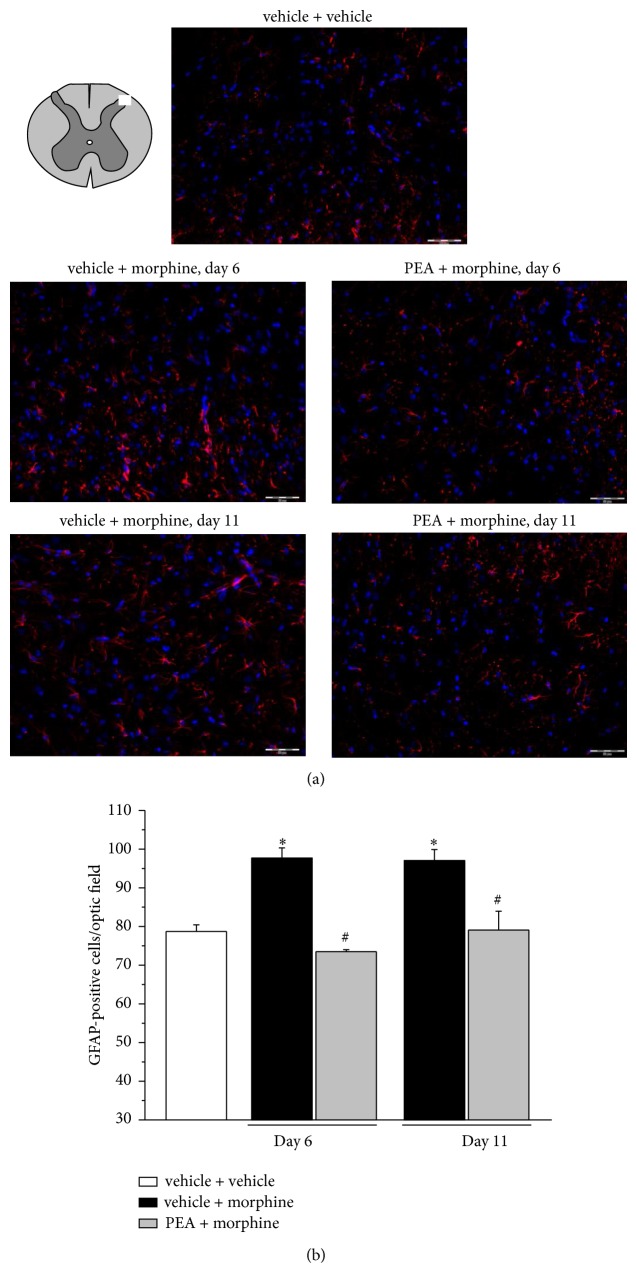
GFAP-positive cell density in the dorsal horn of the spinal cord. 30 mg kg^−1^ PEA s.c. and 10 mg kg^−1^ morphine i.p. were administered daily and immunohistochemical analysis was performed on days 6 and 11; (a) representative images of merged GFAP-labeled astrocyte cells (red), plus DAPI-labeled cell nuclei (blue); scale bar: 50 *μ*m. (b) Quantitative analysis of cellular density was performed evaluating 6 animals for each group. Each value represents the mean ± SEM of 6 rats per group, performed in 2 different experimental sets. ^*^ *P* < 0.05 versus vehicle + vehicle; ^#^ *P* < 0.05 versus vehicle + morphine.

Alkaloid treatment progressively increased the number of Iba1-positive cells in the dorsal horn (Figure 3). On day 6, microglia density was significantly higher in the group vehicle + morphine in comparison to vehicle + vehicle group. PEA fully prevented the morphine-induced microglia activation on day 6 and on day 11 the effect was still significant in comparison to vehicle + morphine (Figure 3). Similar results were obtained when microglia was analyzed by OX42 immunoreactivity (Supplemental information, Figure S1 available online at http://dx.doi.org/10.1155/2014/894732). The expression of GFAP in the dorsal horn is shown in Figure 4. The analysis of GFAP-positive cells reveals a morphine-induced increase in astrocyte cell density on day 6 as well as on day 11 (vehicle + morphine). PEA reduced astrocyte cell number at both time points (Figure 4; PEA + morphine).

**Figure 5:**
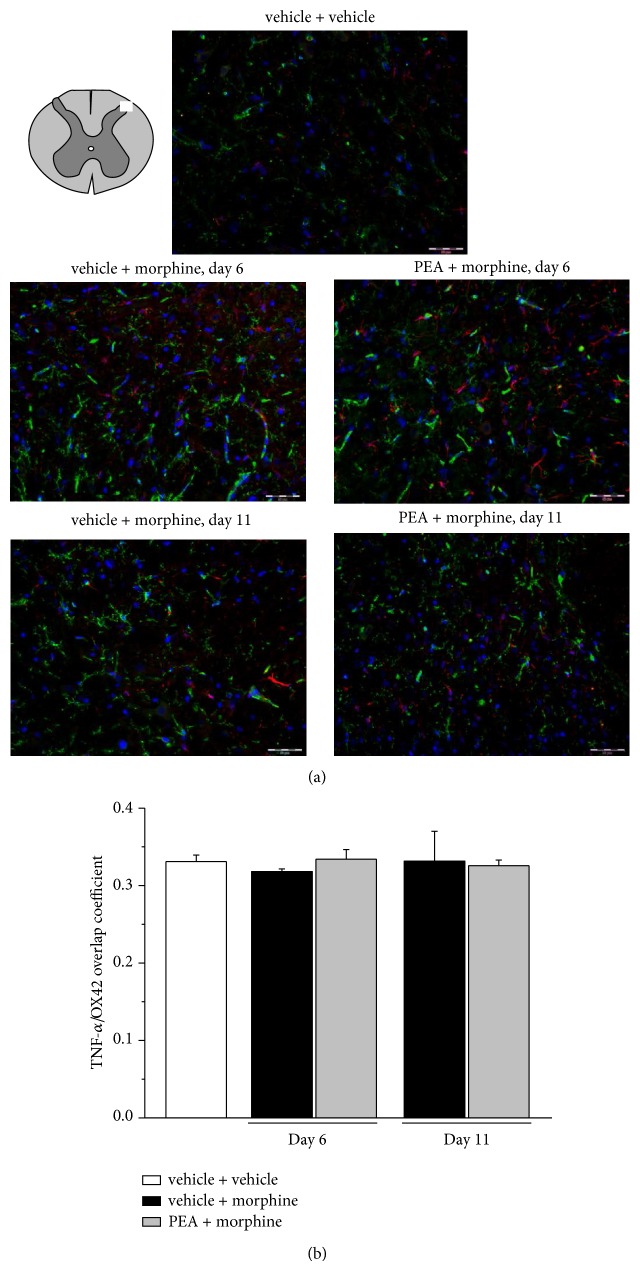
Colocalization of TNF-*α* and OX42 in the dorsal horn of the spinal cord. 30 mg kg^−1^ PEA s.c. and 10 mg kg^−1^ morphine i.p. were administered daily and immunohistochemical analysis was performed on days 6 and 11; (a) representative images of merged TNF-*α* (red), OX42 (green), and DAPI (blue) labeling; scale bar: 50 *μ*m. (b) Quantitative analysis of the overlap coefficient for TNF-*α* and OX42 expression performed evaluating 6 animals for each group. Each value represents the mean ± SEM of 6 rats per group, performed in 2 different experimental sets.

**Figure 6:**
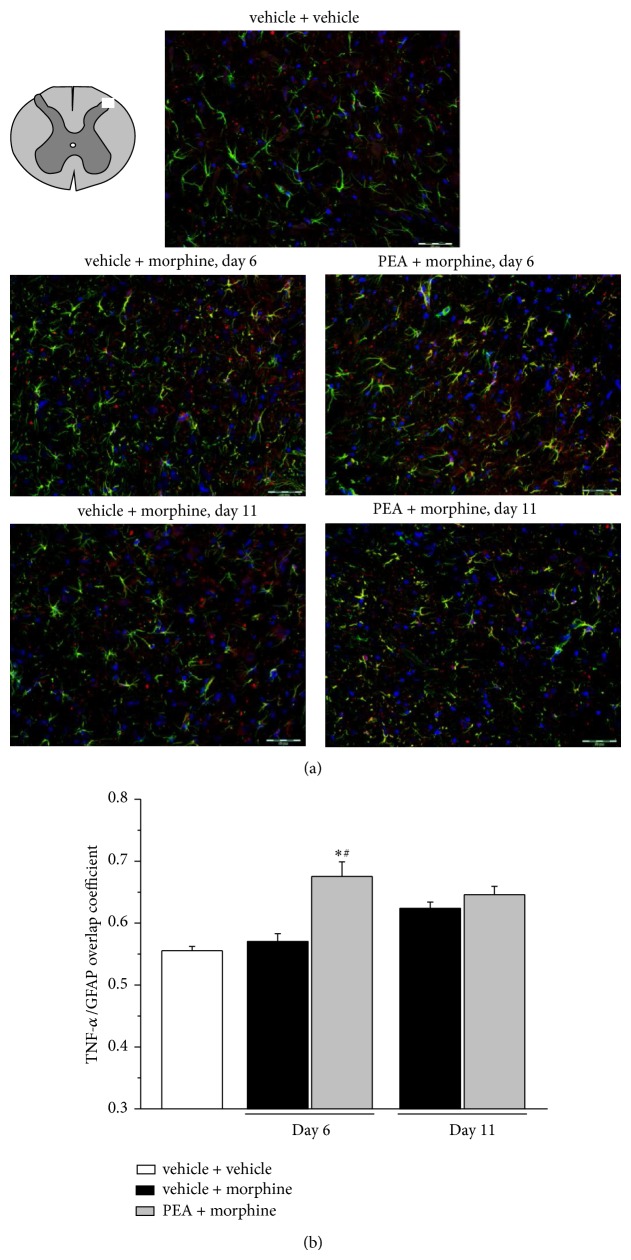
Colocalization of TNF-*α* and GFAP in the dorsal horn of the spinal cord. 30 mg kg^−1^ PEA s.c. and 10 mg kg^−1^ morphine i.p. were administered daily and immunohistochemical analysis was performed on days 6 and 11; (a) representative images of merged TNF-*α* (red), GFAP (green), and DAPI (blue) labeling; scale bar: 50 *μ*m. (b) Quantitative analysis of the overlap coefficient for TNF-*α* and GFAP expression performed evaluating 6 animals for each group. Each value represents the mean ± SEM of 6 rats per group, performed in 2 different experimental sets. ^*^ *P* < 0.05 versus vehicle + vehicle; ^#^ *P* < 0.05 versus vehicle + morphine.

In dorsal horn homogenate TNF-*α* levels were measured (Table 1). Vehicle + morphine treated rats showed a 112% and 168% increase of the cytokine on days 6 and 11, respectively. PEA did not alter this increment. As shown in Figures 5 and 6, TNF-*α* localization was studied by immunohistochemistry. In the spinal dorsal horn of vehicle + vehicle treated rats, TNF-*α* immunoreactivity was scarcely colocalized with the microglial marker OX42 and the overlap coefficient was not modified by treatments (Figure 5). The colocalization of TNF-*α* with GFAP was more evident even though morphine repeated treatment did not alter the value. On the contrary, after 6 days of treatment, PEA (PEA + morphine) significantly increased the overlap between TNF-*α* and GFAP (Figure 6). On day 11, after the development of tolerance also in the PEA group, the cytokine presence in astrocytes decreased to the level of vehicle + morphine group (Figure 6). For all the immunochemical analyses no differences were highlighted in the vehicle + vehicle group on days 6 and 11.

## 4. Discussion

The present data show the property of PEA to double the number of days of morphine treatment efficacy. The pain threshold evaluated by both mechanical and thermal stimuli is still significantly increased by the alkaloid after 10 days of treatment in animals receiving PEA. This result is not influenced by* per se* antinociceptive effects of PEA as shown by pretest values recorded before morphine treatment. This piece is added to the intriguing mosaic of the pain reliever effects of PEA.

Both opioid tolerance and neuropathic pain conditions share features of diminished morphine analgesia, leading to suggestions of a common mechanism [39]. Among complex signaling networks, glial cell modulation emerges in neuropathic pain [40–42] and in antinociceptive tolerance [6] as well as in PEA effects [24].

To the best of our knowledge, this is the first evidence of morphine-induced glial activation characterized by an increase in cell density without consistent morphological alteration of both microglia and astrocytes. This glial profile is evident on day 6 when morphine lacks its antinociceptive properties. PEA prevents the glial cell number increase and prolongs morphine efficacy up to day 10 suggesting a relationship between glial inhibition and attenuation of tolerance. Nevertheless, the preventative effect on the glial density increase is maintained also on day 11 after the onset of tolerance in the group PEA + morphine suggesting a further mechanism based on glia functions. Glial cells activated by morphine secrete large amounts of proinflammatory cytokines including interleukin-1*β* (IL-1*β*), IL-6, and TNF-*α*, ATP, and nitric oxide (NO), accelerating the development of the antinociceptive tolerance [43]. Moreover, IL-1*β*, IL-6, and TNF-*α* have also been shown to oppose acute and chronic opioid analgesia [44]. Glia-derived proinflammatory cytokines inhibit the antinociceptive effect of morphine by sensitizing pain-transmission neurons in animals with morphine tolerance and neuropathic pain [45]. Both central and peripheral administration of the proinflammatory cytokines TNF-*α*, IL-1*β*, and IL-6 facilitate pain transmission [46, 47] and the reduction of the antinociceptive effect of morphine can be reversed by inhibition of glial metabolism, antagonism of IL-1 receptors, and induction of anti-inflammatory cytokine IL-10 expression [6, 48]. In the present study, the expression levels of TNF-*α* in spinal cord tissue homogenate are increased after 6 and 11 days of morphine treatment but PEA does not modify this alteration. The double immunofluorescence analysis of dorsal horn revealed a preferential localization of TNF-*α* astrocyte in comparison to microglia cells and, interestingly, significantly higher TNF-*α* immunoreactivity in astrocytes of PEA-protected rats (day 6) is shown. Since the presence of TNF-*α* in GFAP-positive cells decreases on day 11, PEA seems to be able to delay the cytokine release from astrocyte paralleling with tolerance attenuation. The relevance of astrocyte-released TNF-*α* in tolerance mechanisms was highlighted by Wang et al. [49] which demonstrated, after a 7-day treatment with morphine, the cytokine upregulation in astrocytes by a calcitonin gene-related peptide- (CGRP-) mediated increase of phosphorylated ERK [49]. Furthermore Shen and coworkers [50] confirmed the role of TNF-*α* since etanercept, a recombinant soluble p75 TNF receptor:Fc fusion protein [51], preserved a significant antinociceptive effect of morphine in morphine-tolerant rats suppressing proinflammatory cytokine expression and neuroinflammation in microglia [50]. On the contrary, the glial modulation profile and cytokine levels during the period of morphine efficacy remain to be assessed. Even though some relevant upstream signals, including ceramide and nitroxidative stress [52], were shown further research is necessary to clearly highlight the neuron-glia network in the development of morphine tolerance.

PEA is a naturally occurring amide between palmitic acid and ethanolamine; it is a lipid messenger known to mimic several endocannabinoid-driven actions even though PEA does not bind CB1, CB2, and abn-CBD receptors [53]. So far, numerous actions of PEA on immune cells such as modulation of cytokine release from macrophages, attenuation of leukocyte extravasation, and inhibition of mast cell degranulation have been described [54, 55]. Anti-inflammatory effects of PEA have been associated with peroxisome proliferator-activated receptor- (PPAR-) *α* activation [56]. PPAR-*α*, well known for its role in lipid metabolism, controls transcriptional programs involved in the development of inflammation through mechanisms that include direct interactions with the proinflammatory transcription factors NF-kB and AP1 and modulation of IkB function [57]. The pivotal role of PPAR-*α* in the PEA pharmacodynamic mechanisms has been demonstrated for pain relief [17] as well as for the PEA neurorestorative properties after peripheral nerve injury [20]. PPAR-*α* participates also in the PEA modulation of microglial cells [58]. The involvement of PPAR-*α* in morphine tolerance development is not actually established; on the contrary, the PPAR-*γ* agonist pioglitazone reduced the tolerance to the analgesic effect of morphine [59]. An “entourage effect hypothesis” has also been put forward to account for the pharmacological actions of PEA. Based on an activity enhancement of other endogenous compounds (e.g., the endocannabinoid anandamide [16]), by potentiating their affinity for a receptor or by inhibiting their metabolic degradation [60], PEA may indirectly stimulate the transient receptor potential vanilloid type 1 (TRPV1) and the cannabinoid receptors [24]. Interestingly, morphine is able to modulate endocannabinoid levels. Viganò et al. [61] showed modified brain levels of arachidonoylethanolamide (anandamide, AEA) and 2-arachidonoylglycerol (2-AG) after morphine treatment, and differences were highlighted between compounds depending on the duration of morphine exposure and brain area. In particular, a single morphine injection increased AEA whereas it returned to the basal level after 3 days of treatment [61]. CB1 and opioid receptors are colocalized in brain regions important for the expression of morphine dependence [62] and, finally, compounds that modulate the CB1 receptor systems can modulate the development of morphine tolerance and dependence [63]. Repeated administration of the naturally occurring cannabinoid agonist Δ9-tetrahydrocannabinol or the CB1 receptor agonist CP-55940 attenuates morphine antinociceptive tolerance [63–65]. Cannabinoids act on glia and neurons to inhibit the release of proinflammatory molecules, including IL-1*β*, TNF-*α*, and NO [66, 67], and enhance the release of the anti-inflammatory cytokines IL-4 and IL-10 [68]. In particular, anandamide reduces the release of TNF-*α* from astrocytes [66] and the CB2 receptor stimulation attenuated morphine-induced microglial proinflammatory mediator increases, interfering with morphine effect by acting on the Akt-ERK1/2 signalling pathway [69]. On the other hand, PEA reduces activation of microglia and astrocytes expressing cannabinoid CB2 receptors in mice underwent compressive trauma of spinal cord [26].

## 5. Conclusion

Multiple properties of PEA converge to an interaction with signals evoked by morphine. The evidence of a delayed development of tolerance to the antinociceptive effects of morphine in the presence of PEA suggests a possible application of this endogenous compound in opioid-based therapies.

## Supplementary Material

PEA fully prevented the morphine-induced microglia activation (analyzed by OX42 immunoreactivity) on day 6, and on day 11 the effect was still significant in comparison to vehicle + morphine (Figure S1. OX42-positive cell density in the dorsal horn of the spinal cord. 30 mg kg-1 PEA s.c. and 10 mg kg-1 morphine i.p. were administered daily and immunohistochemical analysis were performed on days 6 and 11. Quantitative analysis of cellular density was performed evaluating 6 animals for each group. Each value represents the mean ± SEM of 6 rats per group, performed in 2 different experimental sets. ∗P<0.05 versus vehicle + vehicle; #P<0.05 versus vehicle + morphine).

## Figures and Tables

**Table 1:** TNF-*α* levels in the spinal cord.

	TNF-*α* levels (pg/mg proteins)		
	vehicle + vehicle	vehicle + morphine	PEA + morphine
	12.2 ± 3.2		
Day 6		25.9 ± 3.8^*^	30.7 ± 5.0^*^
Day 11		32.7 ± 6.4^*^	28.4 ± 3.0^*^

In the dorsal horn of the spinal cord, TNF-*α* levels were measured by ELISA on days 6 and 11. Rats were treated daily i.p. with 10 mg kg^−1^morphine and 30 mg kg^−1^ PEA and compared with vehicle treatment. Each value represents the mean ± SEM of 6 rats per group, performed in 2 different experimental sets. ^*^ *P* < 0.05 versus vehicle + vehicle.

## References

[1] Trescot A., Glaser S. E., Hansen H., Benyamin R., Patel S., Manchikanti L. (2008). Effectiveness of opioids in the treatment of chronic non-cancer pain. *Pain Physician*.

[2] Xu J.-T., Zhao J.-Y., Zhao X. (2014). Opioid receptor-triggered spinal mTORC1 activation contributes to morphine tolerance and hyperalgesia. *Journal of Clinical Investigation*.

[3] Zhou D., Chen M.-L., Zhang Y.-Q., Zhao Z.-Q. (2010). Involvement of spinal microglial P2X7 receptor in generation of tolerance to morphine analgesia in rats. *Journal of Neuroscience*.

[4] Cui Y., Chen Y., Zhi J. L., Guo R. X., Feng J. Q., Chen P. X. (2006). Activation of p38 mitogen-activated protein kinase in spinal microglia mediates morphine antinociceptive tolerance. *Brain Research*.

[5] Cui Y., Liao X.-X., Liu W. (2008). A novel role of minocycline: attenuating morphine antinociceptive tolerance by inhibition of p38 MAPK in the activated spinal microglia. *Brain, Behavior, and Immunity*.

[6] Song P., Zhao Z.-Q. (2001). The involvement of glial cells in the development of morphine tolerance. *Neuroscience Research*.

[7] Mika J. (2008). Modulation of microglia can attenuate neuropathic pain symptoms and enhance morphine effectiveness. *Pharmacological Reports*.

[8] Watkins L. R., Hutchinson M. R., Johnston I. N., Maier S. F. (2005). Glia: novel counter-regulators of opioid analgesia. *Trends in Neurosciences*.

[9] Horvath R. J., Landry R. P., Romero-Sandoval E. A., DeLeo J. A. (2010). Morphine tolerance attenuates the resolution of postoperative pain and enhances spinal microglial p38 and extracellular receptor kinase phosphorylation. *Neuroscience*.

[10] Hameed H., Hameed M., Christo P. J. (2010). The effect of morphine on glial cells as a potential therapeutic target for pharmacological development of analgesic drugs. *Current Pain and Headache Reports*.

[11] Eidson L. N., Murphy A. Z. (2013). Blockade of toll-like receptor 4 attenuates morphine tolerance and facilitates the pain relieving properties of morphine. *The Journal of Neuroscience*.

[12] Raghavendra V., Tanga F. Y., DeLeo J. A. (2004). Attenuation of morphine tolerance, withdrawal-induced hyperalgesia , and associated spinal inflammatory immune responses by propentofylline in rats. *Neuropsychopharmacology*.

[13] Mika J., Osikowicz M., Makuch W., Przewlocka B. (2007). Minocycline and pentoxifylline attenuate allodynia and hyperalgesia and potentiate the effects of morphine in rat and mouse models of neuropathic pain. *European Journal of Pharmacology*.

[14] Lu C.-H., Chao P.-C., Borel C. O. (2004). Preincisional intravenous pentoxifylline attenuating perioperative cytokine response, reducing morphine consumption, and improving recovery of bowel function in patients undergoing colorectal cancer surgery. *Anesthesia and Analgesia*.

[15] Han Y., Jiang C., Tang J. (2014). Resveratrol reduces morphine tolerance by inhibiting microglial activation via AMPK signalling. *European Journal of Pain*.

[16] Calignano A., La Rana G., Giuffrida A., Piomelli D. (1998). Control of pain initiation by endogenous cannabinoids. *Nature*.

[17] LoVerme J., Russo R., La Rana G. (2006). Rapid broad-spectrum analgesia through activation of peroxisome proliferator-activated receptor-alpha. *Journal of Pharmacology and Experimental Therapeutics*.

[18] Hesselink J. M. K. (2012). New targets in pain, non-neuronal cells, and the role of palmitoylethanolamide. *Open Pain Journal*.

[19] Hesselink J. M. K. (2013). Chronic idiopathic axonal neuropathy and pain, treated with the endogenous lipid mediator palmitoylethanolamide: a case collection. *International Medical Case Reports Journal*.

[20] Di Cesare Mannelli L., D'Agostino G., Pacini A. (2013). Palmitoylethanolamide is a disease-modifying agent in peripheral neuropathy: pain relief and neuroprotection share a PPAR-alpha-mediated mechanism. *Mediators of Inflammation*.

[21] Lambert D. M., Vandevoorde S., Diependaele G., Govaerts S. J., Robert A. R. (2001). Anticonvulsant activity of N-palmitoylethanolamide, a putative endocannabinoid, in mice. *Epilepsia*.

[22] D'Agostino G., Russo R., Avagliano C., Cristiano C., Meli R., Calignano A. (2012). Palmitoylethanolamide protects against the amyloid-*β*25-35-induced learning and memory impairment in mice, an experimental model of alzheimer disease. *Neuropsychopharmacology*.

[23] Mazzari S., Canella R., Petrelli L., Marcolongo G., Leon A. (1996). N-(2-Hydroxyethyl) hexadecanamide is orally active in reducing edema formation and inflammatory hyperalgesia by down-modulating mast cell activation. *European Journal of Pharmacology*.

[24] Skaper S. D., Facci L., Giusti P. (2013). Glia and mast cells as targets for palmitoylethanolamide, an anti-inflammatory and neuroprotective lipid mediator. *Molecular Neurobiology*.

[25] Luongo L., Guida F., Boccella S. (2013). Palmitoylethanolamide reduces formalin-induced neuropathic-like behaviour through spinal glial/microglial phenotypical changes in mice. *CNS and Neurological Disorders: Drug Targets*.

[26] Esposito E., Paterniti I., Mazzon E. (2011). Effects of palmitoylethanolamide on release of mast cell peptidases and neurotrophic factors after spinal cordinjury. *Brain, Behavior, and Immunity*.

[27] Esposito E., Impellizzeri D., Mazzon E., Paterniti I., Cuzzocrea S. (2012). Neuroprotective activities of palmitoylethanolamide in an animal model of Parkinson's disease. *PLoS ONE*.

[28] Scuderi C., Esposito G., Blasio A. (2011). Palmitoylethanolamide counteracts reactive astrogliosis induced by *β*-amyloid peptide. *Journal of Cellular and Molecular Medicine*.

[29] Habibi-Asl B., Hassanzadeh K., Charkhpour M. (2009). Central administration of minocycline and riluzole prevents morphine-induced tolerance in rats. *Anesthesia & Analgesia*.

[30] Li J. X., Thorn D. A., Qiu Y., Peng B. W., Zhang Y. (2014). Antihyperalgesic effects of imidazoline I(2) receptor ligands in rat models of inflammatory and neuropathic pain. *British Journal of Pharmacology*.

[31] Di Cesare Mannelli L., Bani D., Bencini A. (2013). Therapeutic effects of the superoxide dismutase mimetic compound MnIIMe_2_DO2A on experimental articular pain in rats. *Mediators of Inflammation*.

[32] Leighton G. E., Rodriguez R. E., Hill R. G., Hughes J. (1988). *κ*-Opioid agonists produce antinociception after i.v. and i.c.v. but not intrathecal administration in the rat. *British Journal of Pharmacology*.

[33] Hargreaves K., Dubner R., Brown F., Flores C., Joris J. (1988). A new and sensitive method for measuring thermal nociception in cutaneous hyperalgesia. *Pain*.

[34] Di Cesare Mannelli L., Pacini A., Bonaccini L., Zanardelli M., Mello T., Ghelardini C. (2013). Morphologic features and glial activation in rat oxaliplatin-dependent neuropathic pain. *The Journal of Pain*.

[35] Di Cesare Mannelli L., Pacini A., Matera C. (2014). Involvement of *α*7 nAChR subtype in rat oxaliplatin-induced neuropathy: effects of selective activation. *Neuropharmacology*.

[36] Tomassoni D., Amenta F., Amantini C. (2013). Brain activity of Thioctic acid enantiomers: *In vitro* and *in vivo* studies in an animal model of cerebrovascular injury. *International Journal of Molecular Sciences*.

[37] Zinchuk V., Zinchuk O., Okada T. (2007). Quantitative colocalization analysis of multicolor confocal immunofluorescence microscopy images: pushing pixels to explore biological phenomena. *Acta Histochemica et Cytochemica*.

[38] Zinchuk V., Grossenbacher-Zinchuk O. (2009). Recent advances in quantitative colocalization analysis: focus on neuroscience. *Progress in Histochemistry and Cytochemistry*.

[39] Mayer D. J., Mao J., Holt J., Price D. D. (1999). Cellular mechanisms of neuropathic pain, morphine tolerance, and their interactions. *Proceedings of the National Academy of Sciences of the United States of America*.

[40] Scholz J., Woolf C. J. (2007). The neuropathic pain triad: neurons, immune cells and glia. *Nature Neuroscience*.

[41] Nativi C., Gualdani R., Dragoni E. (2013). A TRPA1 antagonist reverts oxaliplatin-induced neuropathic pain. *Scientific Reports*.

[42] Di Cesare Mannelli L., Pacini A., Micheli L., Tani A., Zanardelli M., Ghelardini C. (2014). Glial role in oxaliplatin-induced neuropathic pain. *Experimental Neurology*.

[43] Berrios I., Castro C., Kuffler D. P. (2008). Morphine: axon regeneration, neuroprotection, neurotoxicity, tolerance, and neuropathic pain. *Puerto Rico Health Sciences Journal*.

[44] Hutchinson M. R., Coats B. D., Lewis S. S. (2008). Proinflammatory cytokines oppose opioid-induced acute and chronic analgesia. *Brain, Behavior, and Immunity*.

[45] Stefano G. B. (1998). Autoimmunovascular regulation: morphine and ancondamide and ancondamide stimulated nitric oxide release. *Journal of Neuroimmunology*.

[46] Reeve A. J., Patel S., Fox A., Walker K., Urban L. (2000). Intrathecally administered endotoxin or cytokines produce allodynia, hyperalgesia and changes in spinal cord neuronal responses to nociceptive stimuli in the rat. *European Journal of Pain*.

[47] DeLeo J. A., Colburn R. W., Nichols M., Malhotra A. (1996). Interleukin-6-mediated hyperalgesia/allodynia and increased spinal IL-6 expression in a rat mononeuropathy model. *Journal of Interferon & Cytokine Research*.

[48] Johnston I. N., Milligan E. D., Wieseler-Frank J. (2004). A role for proinflammatory cytokines and fractalkine in analgesia, tolerance, and subsequent pain facilitation induced by chronic intrathecal morphine. *Journal of Neuroscience*.

[49] Wang Z., Ma W., Chabot J.-G., Quirion R. (2009). Cell-type specific activation of p38 and ERK mediates calcitonin gene-related peptide involvement in tolerance to morphine-induced analgesia. *The FASEB Journal*.

[50] Shen C.-H., Tsai R.-Y., Shih M.-S. (2011). Etanercept restores the antinociceptive effect of morphine and suppresses spinal neuroinflammation in morphine-tolerant rats. *Anesthesia and Analgesia*.

[51] Scott D. L., Kingsley G. H. (2006). Tumor necrosis factor inhibitors for rheumatoid arthritis. *The New England Journal of Medicine*.

[52] Ndengele M. M., Cuzzocrea S., Masini E. (2009). Spinal ceramide modulates the development of morphine antinociceptive tolerance via peroxynitrite-mediated nitroxidative stress and neuroimmune activation. *Journal of Pharmacology and Experimental Therapeutics*.

[53] Mackie K., Stella N. (2006). Cannabinoid receptors and endocannabinoids: evidence for new players. *AAPS Journal*.

[54] Ross R. A., Brockie H. C., Pertwee R. G. (2000). Inhibition of nitric oxide production in RAW264.7 macrophages by cannabinoids and palmitoylethanolamide. *European Journal of Pharmacology*.

[55] Berdyshev E. V. (2000). Cannabinoid receptors and the regulation of immune response. *Chemistry and Physics of Lipids*.

[56] Lo Verme J., Fu J., Astarita G. (2005). The nuclear receptor peroxisome proliferator-activated receptor-*α* mediates the anti-inflammatory actions of palmitoylethanolamide. *Molecular Pharmacology*.

[57] Glass C. K., Ogawa S. (2006). Combinatorial roles of nuclear receptors in inflammation and immunity. *Nature Reviews Immunology*.

[58] Franklin A., Parmentier-Batteur S., Walter L., Greenberg D. A., Stella N. (2003). Palmitoylethanolamide increases after focal cerebral ischemia and potentiates microglial cell motility. *Journal of Neuroscience*.

[59] de Guglielmo G., Scuppa G., Kallupi M., Stopponi S., Ciccocioppo R. Modulation of PPAR*γ* receptors regulates tolerance to morphine analgesia.

[60] Smart D., Jonsson K.-O., Vandevoorde S., Lambert D. M., Fowler C. J. (2002). ‘Entourage’ effects of N-acyl ethanolamines at human vanilloid receptors. Comparison of effects upon anandamide-induced vanilloid receptor activation and upon anandamide metabolism. *British Journal of Pharmacology*.

[61] Viganò D., Valenti M., Cascio M. G., Di Marzo V., Parolaro D., Rubino T. (2004). Changes in endocannabinoid levels in a rat model of behavioural sensitization to morphine. *European Journal of Neuroscience*.

[62] Navarro M., Chowen J., Carrera M. R. A. (1998). CB1 cannabinoid receptor antagonist-induced opiate withdrawal in morphine-dependent rats. *NeuroReport*.

[63] Fischer B. D., Ward S. J., Henry F. E., Dykstra L. A. (2010). Attenuation of morphine antinociceptive tolerance by a CB1 receptor agonist and an NMDA receptor antagonist: interactive effects. *Neuropharmacology*.

[64] Cichewicz D. L., Welch S. P. (2003). Modulation of oral morphine antinociceptive tolerance and naloxone-precipitated withdrawal signs by oral *δ*9-tetrahydrocannabinol. *Journal of Pharmacology and Experimental Therapeutics*.

[65] Smith P. A., Selley D. E., Sim-Selley L. J., Welch S. P. (2007). Low dose combination of morphine and Δ9-tetrahydrocannabinol circumvents antinociceptive tolerance and apparent desensitization of receptors. *European Journal of Pharmacology*.

[66] Molina-Holgado F., Lledó A., Guaza C. (1997). Anandamide suppresses nitric oxide and TNF-*α* responses to Theiler's virus or endotoxin in astrocytes. *NeuroReport*.

[67] Cabral G. A., Harmon K. N., Carlisle S. J. (2001). Cannabinoid-mediated inhibition of inducible nitric oxide production by rat microglial cells: evidence for CB1 receptor participation. *Advances in Experimental Medicine and Biology*.

[68] Klein T. W., Lane B., Newton C. A., Friedman H. (2000). The cannabinoid system and cytokine network. *Proceedings of the Society for Experimental Biology and Medicine*.

[69] Merighi S., Gessi S., Varani K., Fazzi D., Mirandola P., Borea P. A. (2012). Cannabinoid CB2 receptor attenuates morphine-induced inflammatory responses in activated microglial cells. *British Journal of Pharmacology*.

