# Supplementary figures and images for: Delay of Morphine Tolerance by Palmitoylethanolamide
Source: Biomed Res Int. 2015 Mar 22;2015:894732. doi: 10.1155/2015/894732 (PMC4385605; doi:10.1155/2015/894732)

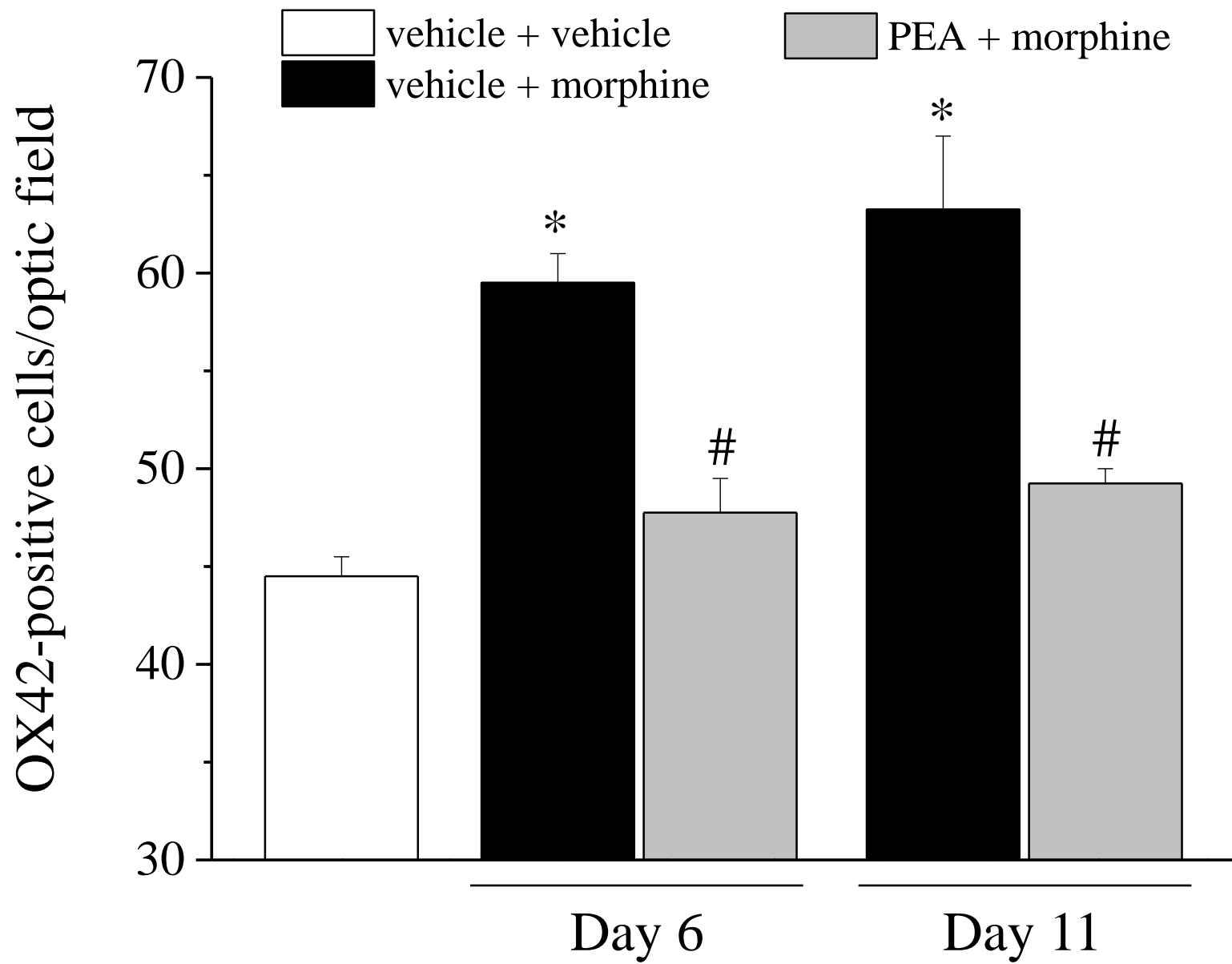

Supplement: Supplementary file 1 — PEA fully prevented the morphine-induced microglia activation (analyzed by OX42 immunoreactivity) on day 6, and on day 11 the effect was still significant in comparison to vehicle + morphine (Figure S1. OX42-positive cell density in the dorsal horn of the spinal cord. 30 mg kg-1 PEA s.c. and 10 mg kg-1 morphine i.p. were administered daily and immunohistochemical analysis were performed on days 6 and 11. Quantitative analysis of cellular density was performed evaluating 6 animals for each group. Each value represents the mean ± SEM of 6 rats per group, performed in 2 different experimental sets. ∗P<0.05 versus vehicle + vehicle; #P<0.05 versus vehicle + morphine). [file 894732.f1.pdf]
